# Correlation between the major components of *Phyllanthus amarus* and *Phyllanthus urinaria* and their inhibitory effects on phagocytic activity of human neutrophils

**DOI:** 10.1186/1472-6882-14-429

**Published:** 2014-11-01

**Authors:** Ibrahim Jantan, Menaga Ilangkovan, Yuandani, Hazni Falina Mohamad

**Affiliations:** Drug and Herbal Research Center, Faculty of Pharmacy, Universiti Kebangsaan Malaysia, Jalan Raja Muda Abdul Aziz, Kuala Lumpur, 50300 Malaysia

**Keywords:** *Phyllanthus amarus*, *Phyllanthus urinaria*, Immunomodulatory effects, Phagocytosis, Phyllanthin, Hypophyllanthin, Geraniin, Corilagin

## Abstract

**Background:**

Recently, we have highlighted the immunomodulatory activity of the standardized extracts of *Phyllanthus amarus* and *P. urinaria.* The present study was carried out to correlate between the prevalent constituents of the herbs and their inhibitory effects on phagocytic activity of human neutrophils.

**Methods:**

The compounds, gallic acid, ellagic acid, corilagin, geraniin, phyllanthin and hypophyllanthin were identified and quantitatively analyzed in the extracts of *Phyllanthus amarus* and *P. urinaria* obtained from Malaysia and Indonesia by using a validated reversed phase high performance liquid chromatography (RP-HPLC) method. The standardized extracts and the pure compounds were evaluated for their effects on chemotaxis, β2 integrin (CD18) expression, phagocytosis and chemiluminescence of human phagocytes. Chemotactic activity was assessed using the Boyden chamber technique, inhibition of CD18 expression and phagocytic ability were tested with the aid of flow cytometry, while effect on the respiratory burst was investigated using a luminol-based chemiluminescence assay.

**Results:**

All plant extracts strongly inhibited migration of the phagocytes with the Malaysian *P. amarus* depicting the highest inhibitory activity. Amongst the compounds tested, geraniin demonstrated the strongest inhibitory activity on chemotaxis of polymorphonuclear leukocytes (PMNs) and monocytes with IC_50_ values of 1.09 and 1.69 μM, respectively, which were lower than that of ibuprofen. All plant extracts and pure compounds exhibited high inhibitory activity on the oxidative burst of zymosan and PMA stimulated leukocytes. Geraniin and corilagin exhibited exceptionally strong inhibition on the reactive oxygen species (ROS) activity with IC_50_ values lower than aspirin. The plant extracts exhibited moderate inhibition of *E. coli* uptake by monocytes but weak effect on PMNs. Of all the compounds, phyllanthin at 50 μg/mL exhibited the highest engulfment inhibitory activity with percentage of phagocytizing cells of 14.2 and 27.1% for PMNs and monocytes, respectively. All plants and compounds tested possessed weak effect on CD18 expression on leukocytes except for hypophyllanthin and phyllanthin which exhibited significant inhibitory effect.

**Conclusion:**

The strong inhibition of the extracts on the phagocytic activity of neutrophils was due to the presence of their major constituents especially geraniin, corilagin, phyllanthin and hypophllanthin which were able to modulate the innate response of phagocytes at different steps.

## Background

Immune system is designed to protect the host by destroying and eliminating the invading pathogen. It requires the timely interplay of a number of different types of cells within a specific microenvironment to maintain the immune homeostasis [1]. Human immune response is divided into two main categories which include innate immunity, initiated by phagocytosis of pathogens by phagocytic cells and this orchestrates the adaptive immune system [2–4]. The two types of circulating phagocytes, neutrophils and monocytes, are the main contributors to innate immune system. In response to invasive bacterial infection, circulating phagocytes perform a number of specific activities, including migration of phagocyte cells to the site of infection, adherence to blood vessel walls, transmigration into injured tissues, phagocytosis, degranulation, and reactive oxygen species production [5]. Chemotaxis is referred to the migration of phagocytes to the site of infection upon the activation by either endogeneous chemoattractants such as interleukin 8 (IL-8), leukotriene B4 (LTB4), platelet activating factor (PAF) or exogeneous chemoattractants such as formyl methionyl-leucyl-phenylalanine (fMLP) derived from bacterial products [6–8].

Rapid regulation of adhesive interactions between phagocytes and vessel wall are essential in proper recruitment of leukocytes and defensive against invasive pathogens. Interactions between leukocytes and endothelial cells are largely mediated by selectin family of receptors and integrins. CD11/CD18 antigen complex have been reported as an important group of adhesion molecule involved in granulocyte interaction with other cells as well as with antigens [9]. The interaction between phagocyte and ligand at the site of infection leads to a sequential event known as oxidative burst to kill the microbial intruders [10, 11]. Oxidative burst involves the increased oxygen consumption and generation of highly reactive superoxide anion radical (O_2_^−^) and hydroxyl radicals (OH^-^) and hydrogen peroxides (H_2_O_2_). Despite its host defense, exaggerated function of human phagocyte can also be involved in cell and tissue damage associated with severe inflammatory reactions and autoimmune disorders [12]. Thus, modulation of phagocytic activity through different pathways plays an essential role in keeping them in a highly prepared state for any threat it may encounter [13].

In this context, the utilization of medicinal plants and their active components as immunomodulators to substitute conventional therapy has gained momentum in recent years [14–18]. However, little is known about the immunomodulatory activity of Malaysian medicinal plants. The plants belonging to the genus *Phyllanthus* (family: Euphorbiaceae) are widely distributed in tropical and subtropical countries which have long been used in folk medicine for the treatment of liver, asthma, kidney and bladder problems and diabetes [19]. Besides, the anti-viral properties of some *Phyllanthus* species, mainly against hepatitis B have extended the medicinal properties of these plants in traditional medicine [20]. Amongst all the species of *Phyllanthus*, *P. amarus* has gained a remarkable interest in past few decades due to its variety of organic compounds of medicinal importance including flavonoids, ellagitannins, alkaloids, polyphenols, lignans and triterpenes [21, 22] *. P. urinaria* is yet another species of *Phyllanthus* which has been reported to possess various biological activities including anti-tumor, anti-angiogenic, anti-inflammation, anti-hypertensive, anti-microbial and anti-viral activities [23–25]. The biosynthesis of secondary metabolites of these plants has been reported to vary among plants grown in different environmental factors [26, 27]. Our previous studies on *Phyllanthus amarus* and *P. urinaria* for their phagocytic properties have revealed that the standardized methanol extracts of these plants have the potential to exhibit strong immunomodulatory effect on human neutrophils [28]. In many previous studies, *Phyllanthus* species have been reported for their wide spectrum of pharmacological activities [29, 30]. However, investigations to correlate the chemical constituents of the plants with their biological and pharmacological effects were seldom reported. The present study was carried out to investigate the effects of the major components of the 80% ethanol extracts of *P. amarus* and *P. urinaria* on phagocytic activity of human neutrophils in an effort to correlate the effectiveness of the plants with those of their components.

## Methods

### Chemicals and reagents

Serum opsonized zymosan A (*Saccharomyces cerevisiae* suspensions and serum), lipopolysaccharide (LPS), Fluorescein isothiocyanate (FITC)-labelled opsonized *Escherichia coli,* luminol (3-aminophthalhydrazide), phosphate buffer saline tablet (PBS), Hanks Balance Salt Solutions (HBSS), Ficoll, Hanks Balance Salt Solution (HBSS), N-formyl-methionylleucyl-phenylalanine (fMLP), Tryphan blue, MTT reagent, aspirin (purity 99%), ibuprofen (purity 99%), phorbol 12-myristate 13-acetate (PMA),and dimethylsulfoxide (DMSO) were purchased from Sigma (St Louis, MO, USA). Fetal calf serum was obtained from PAA Laboratories (USA). CD18-FITC (anti-LFA-1β), isotype-matched Immunoglobulin-FITC (IgG1-FITC) and FACS lysing solution were obtained from Becton Dickinson, USA. Chemiluminescence measurements were carried out on a Luminoskan Ascent luminometer (Thermo Scientific, UK). fMLP was stored as a stock solution of 10^−8^ M in DMSO at −80°C and diluted in Hanks solution, prior to assay. Phagocytic kit was purchased from Oregen Pharmagen, USA. Haematoxylin and xylene for staining were obtained from BDH, UK. A Boyden 48-well chamber with a 3 μm and 5 μm polycarbonate membrane filter separating the upper and lower compartments was purchased from Neuro Probe (Cabin John, MD, USA). Phyllanthin and hypophyllanthin (isolated based on method described in previous study with purity >98%) [28]. Whereas gallic acid, ellagic acid, corilagin and geraniin (purity >98%) were purchased from ChromaDex (CA, USA). Methanol HPLC grade, acetonitrile HPLC grade, and, trifluoroacetic acid AR grade were obtained from E-Merck. Phagotest kit was obtained from Glycotope Technology, Germany. The flow cytometer BDFACS Canto II equipped with 488 nm argon-ion laser was used. A CO_2_ incubator (Shell Lab, USA), light microscope, and high-performance liquid chromatograph (Waters 2998) (Leitz Watzler, Germany) were also used in this assay.

### Plant materials and preparation of extracts

The whole plants of *Phyllanthus amarus* and *P. urinaria* were obtained from Marang, Kuala Terengganu, Malaysia and Tanjung Anom, Northern Sumatra, Indonesia between February and June 2012. The plants were identified by Dr Abdul Latif Mohamad of Faculty of Science and Technology, Universiti Kebangsaan Malaysia (UKM), and voucher specimens (*P. amarus* UKMB 30078 and *P. urinaria* UKMB 30077) were deposited at the Herbarium of UKM, Bangi, Malaysia. No specific permissions were required for the collection of plant samples from Marang, Kuala Terengganu, Malaysia and Tanjung Anom, Northern Sumatra, Indonesia. The collection of plant samples did not involve endangered or protected species and the study was carried out at the Drug and Herbal Research Centre, Faculty of Pharmacy, UKM. The aerial parts of *P. amarus* and *P. urinaria* (100 g each) were ground and extracted with 80% EtOH (3 × 3 L) at room temperature for 72 h and filtered through Whatman No.1 filter paper ( Whatman, England). The filtrate was collected and excess solvent was evaporated under reduced pressure using rotary evaporator at 55°C to dryness to obtain dark yellowish green ethanol extract of *P. amarus* (Malaysia, 10.8 g, 10.8%; Indonesia, 10.1 g, 10.1%) and *P.urinaria* (Malaysia, 9.68 g, 9.7%; Indonesia, 7.48 g, 7.5%).

### Quantitative determination of the major components of plant extracts by HPLC

One hundred mg each of the 80% ethanol extracts of *Phyllanthus amarus* and *P. urinaria* was dissolved into 10 mL of MeOH and 1 mg each of the reference standards (gallic acid, ellagic acid, corilagin, geraniin, phyllanthin and hypophyllanthin) in 1 mL of MeOH were ultra sonicated at ambient temperature for 10 min and filtered through 0.45 *μ*m Millipore Millex PTFE membrane (Maidstone, Kent, UK). Phyllanthin and hypophyllanthin (isolated based on method described in previous study with purity >98%). Whereas gallic acid, ellagic acid, corilagin and geraniin (purity >98%) were purchased from ChromaDex (CA, USA). The diluted solutions of the extracts and the reference standards were analyzed separately by HPLC using the following conditions: Column: Reverse Phase, C-18 column (250 mm × 4.6 mm i.d., 5 *μ*m, Xbridge, Waters, Ireland), and detector: PDA (Waters 2998) of wavelength ranging from 205 to 270 nm. For the analysis of the extracts and standard solutions of phenolic compounds (gallic acid, ellagic acid, corilagin, geraniin) gradient elution was used with acetonitrile 99.99% (solvent A) and orthophosphoric acid 0.2% (solvent B) as mobile phase at a flow rate of 0.6 mL/min with the following procedure; 5-70% A (0 to 15 min), 70 to 95% A (15 to 30 min) and pump A had been hold at 95% for 20 min. Corilagin was used as external standard for the identification and quantification of the phenolic compounds. Identification and quantification of the lignans (phyllanthin and hypophyllanthin) were carried out based on the chromatographic condition as described in our previous study [28]. The identification of each compound was carried out by comparing the retention times and UV-Vis spectra of the peaks with those obtained by the injection of the standards. The calibration curves were plotted with five concentrations each of the standard solution of compounds versus the areas under the peaks. The standard curves equations obtained from each compounds were used to quantify the compounds in the extracts.

### Validation procedures for HPLC analysis

Validation of the HPLC method was carried out by determination of linearity, precision, limits of quantification (LOQ) and detection (LOD). LOD and LOQ were calculated based on the standard deviation (σ) of the responses of the lowest concentration in calibration curve of six runs (n =5) and the slope of the calibration curves of external standards, respectively. Correlation coefficient (R^2^) was calculated from the calibration curves and linearity was evaluated by linear calibration analysis. Intra-assay and inter-assay validation were performed to determine the precision of the method. Separately, one concentration of extracts (10 mg/mL) and reference compounds (1 mg/mL) were injected three times in one day and on three different days. LOD and LOQ were calculated from the RSD and slope (*S*) of the calibration curves using equations: LOD =3.3 × (RSD/*S*) and LOQ =10 × (RSD/*S*).

### Isolation of human polymorphonuclear leukocytes and monocytes

Ten mL of fresh venous blood was obtained in heparin-containing tubes by aseptic vein puncture from healthy human volunteers who fulfilled the following inclusion criteria: non-smoker, fasted overnight and did not take any medicine or supplements. The whole blood was then aliquot into falcon tubes and equal amount of dextran and PBS were added and the mixture was left for 45 min at room temperature (26°C ±2) for sedimentation. The PMN isolation was performed by using a modified method of Oh et al. [31]. The supernatant was centrifuged by Ficoll-gradient separation and then washed twice with cold distilled water for the lysis of red blood cells. PMN cells sedimented at the bottom of the tubes. Whereas the isolation of monocytes was performed by using a modified method of Gmelig et al. [32]. Briefly, 10 mL of venous blood from healthy individual was diluted (1:1) with physiological saline before being applied to the gradient. Diluted blood was then carefully layered on lympho prep and centrifuged at 400 × g for 20 min. After centrifugation the mononuclear cells formed a distinct band at the medium interface and was slowly removed using pasteur pipette without removing the upper layer. The fraction was diluted with PBS and centrifuged at 250 × g for 10 min for the purification of cells. These cells were then suspended in PBS and cell suspension were counted using haemocytometer and number of cells were adjusted to a final concentration of 1 × 10^6^ cells/mL. The use of human blood was approved by the Human Ethical Committee, Faculty of Medicine, Universiti Kebangsaan Malaysia (approval number FF/2012/Ibrahim/23-May/432-May 2012–August 2013). Written informed consent was obtained from individual human volunteer participating in this study prior to blood collection.

### Cell viability

Cytotoxicity of *P. amarus* and *P. urinaria* extracts and standard compounds on monocytes was measured by using MTT [33] while viability of PMNs was determined by the standard trypan blue exclusion method [34]. For the MTT assay, one hundred μL sample of five different concentrations (6.25 - 100 μg/mL) of the samples were filled into a 96 well round bottom microplate containing 100 μL cells suspension in RPMI 1640 10% FCS. The plates were incubated for 24 h at 37°C and 5% CO_2._ Twenty μl of MTT reagent (1 mg/mL) was added into each well and incubated for 4 h at 37°C and 5% CO_2_. The supernatant removed and the formazan produced by cells in each well was dissolved in DMSO and the optical densities (OD) were measured by ELISA readers at 570 nm [33]. The standard trypan blue exclusion assay was carried out by incubating neutrophils (1 × 10^6^/mL) with 6.25 or 100 μg/mL of samples and 3.125 to 100 μg/mL of pure compounds in triplicate at 37°C for 2 h. Cell death was indicated by the blue dye uptake and percentage viability was calculated from the total cell counts [34].

### Chemiluminescence assay

Chemiluminescence assays were carried out as described by Jantan et al. [34]. Briefly, 25 *μ*L diluted whole blood or 25 *μ*L monocytes (adjusted to 1 × 10^6^) or 25 *μ*L PMN (adjusted to 1 × 10^6^) suspended in HBSS^++^were incubated with 25 *μ*L of sample with different concentrations ranging from 6.25 to 100 *μ*g/mL in 96 well flat bottom microplate. Inducement of the cells was with 25 *μ*L of opsonized zymosan or PMA. This was followed by the addition of 25 *μ*L of luminol (1× 10^5^ M) and the final volume of each well was adjusted to 200 μL with HBSS^++^. The microplate was then incubated at 37°C for 50 min in the thermostatically controlled chamber of the luminometer. The control wells contained 0.6% DMSO, HBSS^++^, luminol without sample and aspirin, a non-steroidal antiinflammatory drug (NSAID), was used as a standard drug. The luminometer results were monitored as chemiluminescence RLU (reading per luminometer unit) with peak and total integral values set with repeated scans at 30 s intervals and 1 s points measuring time.

### Chemotaxis assay

The assay was performed using a modified 48-well Boyden chamber with formyl-methionylleucyl-phenylalanine (fMLP) a bacterial peptide as a chemoattractant, as previously described by Jantan et al. [34]. Aliquots of 25 *μ*L of fMLP (10 − 8 M) were added to the wells in lower chamber. Serial dilutions of 5 *μ*L of each extract (6.25–100 *μ*g/mL) were added to the upper chamber containing 45 *μ*L PMNs (1 × 10^6^ cells per mL) or 45 *μ*L monocytes (1 × 10^6^ cells per mL) suspended in HBSS^++^. The final concentrations of the samples in the mixture were adjusted to 10, 5, 2.5, 1.25, and 0.625 *μ*g/mL. The control wells contained the chemoattractant buffer HBSS^++^ and DMSO (1:1). The cells were then incubated for one h at 37°C in a CO_2_ incubator. Haematoxylin and xylene were used to fix and stain migrated cells which had adhered to the distal part of the filters. The cell migration distance was measured by using a light microscope. Ibuprofen was used as a standard drug.

### Phagocytic assay

The assay was performed according to the protocol given by the manufacturer (ORPEGEN Pharma). Briefly, 100 *μ*L heparinized peripheral whole blood was incubated with 20 *μ*L pre-cooled FITC-labelled opsonized *E. coli* and 20 *μ*L of test samples (extracts: 6.25 and 100 *μ*g/mL; pure compounds: 3.125 and 50 *μ*g/mL) in a closed shake water bath at 37°C for 10 min. Positive control was without test sample under similar condition while the negative control was without test sample and remained on ice. At the end of the incubation time ice-cold quenching solution was added to the mixture to quench phagocytosis. Subsequently lysing solution was added for lysis of red blood cells and fixation of leukocytes. Cells were then washed twice and resuspended in 200 *μ*L of DNA staining solution for cytometric discrimination of bacteria during leukocyte analysis. The phagocytic ability of neutrophils and monocytes was determined by flow cytometry using the blue-green excitation light (488 nm argon-ion laser). Live populations were gated by the software program in the scatter diagram (FCS versus SSC). Phagocytic activity was determined as the percentage of phagocytizing neutrophils and monocytes.

### CD18 integrin expression of leukocytes

The CD18 integrin expression assay was performed using a modified method of Mazzone et al. [35]. Peripheral blood was obtained from heparinized venous blood of normal voluntary donors. Aliquots (100 μL) of whole blood were then incubated in the presence or absence of samples with different concentrations at 37°C for 30 min. Thereafter, cells were stimulated with LPS (0.25 μg/mL) for 90 min at 37°C in a CO_2_ incubator. Then, all the tubes were simultaneously transferred into ice bath to stop the reaction. Subsequently 10 μL of CD18-FITC or Immunoglobulin G-FITC (negative control) was added into the mixture. All the tubes were incubated for 60 min on ice. FACS lysing solution was added into each tube and incubated in dark for 20 min for the lysis of red blood cells. The tubes were centrifuged at 250 g for 5 min at 4°C and supernatant was removed from the tubes and cells were washed with PBS (3×). 500 μl of PBS was added into each tube and expression of adhesion molecules was determined by flow cytometry and compared with adhesion molecule expression of untreated cells. Neutrophils and monocytes were discriminated in terms of forward and side scatter. Forward scatter is correlated to cell size, whereas side scatter is related to cell granularity. The specific mean fluorescence intensity for cells stained by each antibody is reported as the ratio of the CD18 to that of the negative control antibody. For each sample, 10,000 cells were analyzed.

### Statistical analysis

All the data are presented as means ± standard error median (SEM) from triplicate experiments and were analyzed using statistical package for social sciences (SPSS) software version 20.0. The IC_50_ values were calculated using Graph PAD Prism 6 Analysis software. Data were analyzed using a one-way analysis of variance (ANOVA)for multiple comparisons. *P*< 0.05 was considered to be statistically significant.

## Results and discussion

Quantitative determination of the major components of the 80% ethanol extracts of *Phyllanthu*s *amarus* and *P. urinaria.*

**Figure 1:**
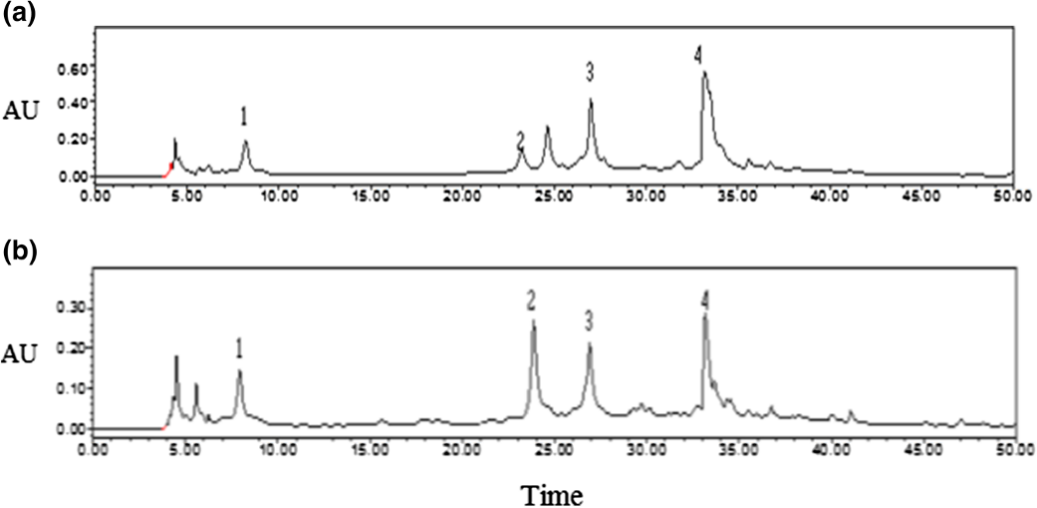
**Representative HPLC chromatograms of (a)** ***P. amarus*** **(Mal), (b)** ***P. urinaria*** **(Mal) for identification and quantification of gallic acid (1) at RT 8.172 min, geraniin (2) at RT 23.694 min,corilagin (3) at RT 26.436 min, ellagic acid (4) at RT 33.529 min at the wavelength of 270 nm. See Table** 2 **for legends.**

The chromatograms of the reversed-phase HPLC column of the 80% ethanol extracts of *P. amarus* and *P. urinaria* showed four major peaks of gallic acid, geraniin, corilagin and ellargic acid, corresponding to retention times at 8.172, 23.694, 26.436 and 33.529 min, respectively (Figure 1). The quantitative determination of the lignans, phyllanthin and hypophyllanthin, their precision, specificity, quantitation limit and detection limit have been reported in our previous study [28]. The identification of gallic acid (GA), ellagic acid (EA), corilagin (Cor) and geraniin (Ger) was carried out by comparing with the retention times of standard solutions of these compounds. The phenolic compounds identified in the extracts were quantified by using corilagin as external standard. The amounts of the major compounds are shown in Table 1. The calibration curve plotted for standard solution of corilagin over the concentration range of 62.5–500 *μ*g/mL showed a correlation coefficient (*r*^2^) of 0.9958. The reproducibility of the results was confirmed by results of relative standard deviation (RSD %) of mean area under peak 1.81% and mean of retention time values 0.93% for interday assay, whereas the % RSD values for intra-assay precision of peak area and retention time were 3.68 and 1.09%. Limit of detection (LOD) and limit of quantification (LOQ) of corilagin were found to be 1.86 and 5.65 ng/mL, respectively.

**Table 1:** **Amount of major compounds found in** ***Phyllanthus amarus*** **and** ***P.urinaria (*** **μ** ***g*** **/mL) obtained from HPLC quantification analysis**

Species	Phyllanthin	Hypophyllanthin	Gallic acid	Ellagic acid	Corilagin	Geraniin
PA (Mal)	103.50	114.00	163.30	601.29	313.41	170.49
PA (Ind)	79.10	121.85	142.92	219.06	413.42	234.52
PU (Mal)	16.80	19.50	103.48	231.34	159.37	173.17
PU (Ind)	1.9	3.91	93.05	346.12	379.23	199.50

PA: *Phyllanthus amarus*, PU: *Phyllanthus urinaria,* Mal: Malaysia, Ind: Indonesia.

Quantitative determination of the major compounds by HPLC indicated that *P. amarus* from Malaysia contained the highest amounts of phyllanthin (103.5 μg/mL), gallic acid (163.3 μg/mL) and ellagic acid (601.29 μg/mL), while *P. amarus* from Indonesia contained the highest amount of hypophyllantin (121.85 μg/mL). Corilagin (379.23 μg/mL) was found in highest concentration in the extract of *P. urinaria* from Indonesia. The variations in the amounts of the major compounds in the plants of similar species collected from different geographical locations were partly due to the different environmental factors related to altitude and genetic adaptation of the populations growing at different altitudes to specific environment [26].

### Chemotaxis assay

**Figure 2:**
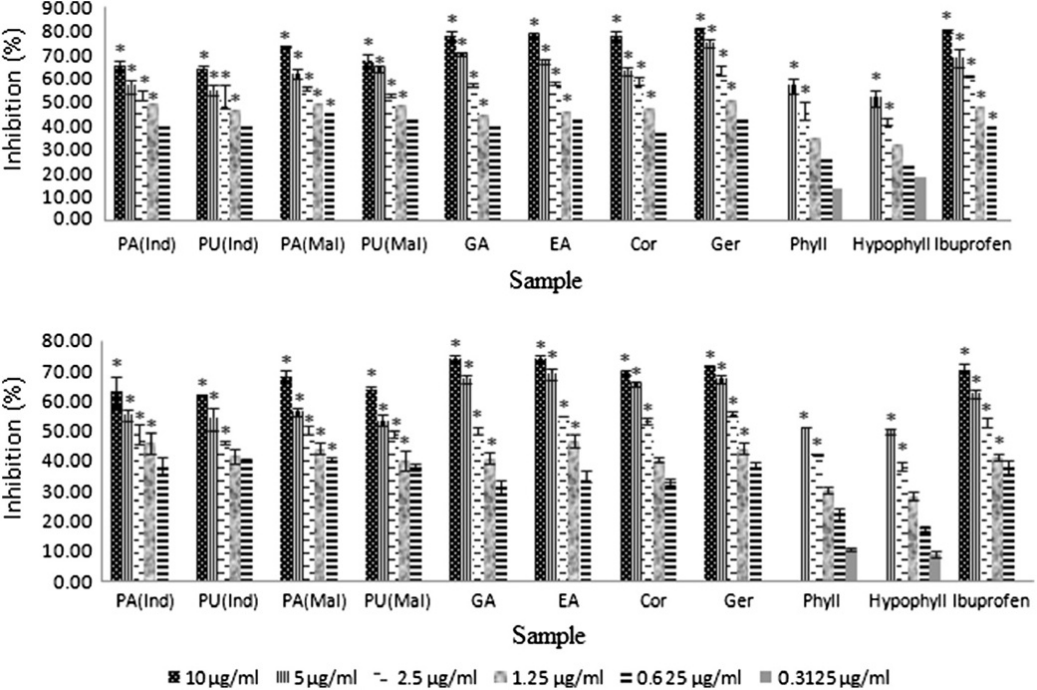
**Percentage inhibition (%) of** ***Phyllanthus*** **sp. and their major compounds on PMN and monocytes chemotaxis.** Data are mean ± SEM (*n* =3). Significance of differences with respective control: *P< 0.05. See Table 2 for legends.

The cell viability test was carried out using trypan blue and MTT assays for PMNs and monocytes, respectively, to evaluate the cytotoxicity of extracts of *Phyllanthus amarus* and *P. urinaria* as well as the standard compounds. The extracts and the standard compounds at concentrations ranging from 6.25 to 100 μg/mL showed more than 95% of cell viability except for phyllanthin and hypophyllanthin which possessed cell viability >95% at concentrations ranging from 0.3125 to 5 μg/mL. The results indicate that leucocytes were viable in the reaction mixtures in the presence of the extracts and the compounds which were non toxic and could potentially modulate the cellular immune response. The inhibitory activities of the extracts and standard compounds at the serial dilutions of 10 to 0.625 *μ*g/mL, on the migration of PMNs towards the chemoattractant (fMLP), were determined and their percentage inhibitions (%) were shown in Figure 2. Chemoattractant buffer (DMSO and HBSS, 1:1 ratio) was used as a control and ibuprofen was used as a positive control. Chemotactic activity of the extracts of *P. amarus* and *P. urinaria* from both Malaysia and Indonesia on human phagocytes in response to N-formylmethionyl-leucylphenylalanine (fMLP) was suppressed in a concentration-dependent manner. Amongst the extracts*, P. amarus* from Malaysia depicted the most potent inhibitory activity on PMNs and monocytes migrations with IC_50_ values of 1.22 and 1.44 *μ*g/mL, respectively (Table 2). Amongst the major compounds identified in *P. amarus* and *P. urinaria*, geraniin, corilagin and ellagic acid demonstrated lower IC_50_ values than that of ibuprofen, with geraniin being the strongest inhibitor with IC_50_ values of 1.09 and 1.69 μM against PMNs and monocytes, respectively. Ibuprofen was used in previous study as a positive control and it was found to be the most effective NSAIDS in blocking the migration of PMNs [36]. There was an inversed correlation between the amount of phenolic compounds and the chemotactic activity of fMLP stimulated phagocytic cells. This is in congruity with previous studies which indicated that the presence of galloyl moeties in phenolics contribute to the inhibition of migration of fMLP stimulated migration of phagocytes by potent inhibition of activity of enzyme elastase [37, 38].

**Table 2:** **IC**_**50**_ **values (** ***μ*** **g/mL) of ROS inhibitory and chemotaxis activities of** ***Phyllanthus amarus*** **and** ***P. urinaria*** **and their major compounds on phagocytes (mean ± SEM,** ***n*** **=3)**

Sample	Chemotaxis		Chemiluminescence					
	fMLP		Zymosan			PMA		
	PMNs	MNCs	Whole Blood	PMNs	MNCs	Whole Blood	PMNs	MNCs
PA (Mal)	1.22 ± 0.10	1.44 ± 0.80	0.95 ± 0.24	0.58 ± 1.80	0.10 ± 0.17	1.24 ± 0.98	0.42 ± 0.75	0.56 ± 0.30
PA (Ind)	1.84 ± 0.01	2.35 ± 0.23	8.14 ± 1.71	0.12 ± 0.05	2.19 ± 2.55	6.02 ± 1.33	1.34 ± 0.59	1.27 ± 0.80
PU (Mal)	1.46 ± 0.10	2.84 ± 0.30	1.52 ± 0.53	1.00 ± 1.21	1.23 ± 0.88	1.98 ± 1.09	0.45 ± 0.23	0.26 ± 0.12
PU (Ind)	2.21 ± 0.90	2.92 ± 0.14	9.74 ± 1.09	1.64 ± 0.58	0.36 ± 0.29	4.80 ± 1.80	1.34 ± 1.18	1.06 ± 0.31
GA	1.40 ± 0.02 (8.22 ± 0.15)	2.00 ± 0.03 (11.75 ± 0.23)	1.70 ± 0.03 (9.99 ± 0.12)	0.43 ± 1.02 (2.53 ± 0.10)	1.29 ± 0.87 (7.58 ± 0.50)	0.87 ± 0.12 (5.11 ± 0.15)	0.48 ± 0.01 (2.82 ± 0.15)	1.28 ± 0.01 (7.52 ± 0.12)
EA	1.33 ± 0.07 (4.40 ± 0.12)	1.81 ± 0.12 (5.96 ± 0.12)	1.36 ± 0.08 (4.50 ± 0.16)	0.56 ± 0.02 (1.85 ± 0.35)	0.45 ± 0.09 (1.49 ± 0.07)	0.83 ± 0.03 (2.75 ± 0.07)	0.31 ± 0.07 (1.02 ± 0.06 )	0.75 ± 0.12 (2.48 ± 0.06 )
Ger	1.04 ± 0.12 (1.09 ± 0.23 )	1.61 ± 0.14 (1.69 ± 0.05)	0.34 ± 0.30 (0.36 ± 0.12 )	0.18 ± 0.07 (0.18 ± 0.02)	0.37 ± 0.41 (0.38 ± 0.07)	0.19 ± 0.12 (0.21 ± 0.01)	0.12 ± 0.04 (0.13 ± 0.01)	0.36 ± 0.61 (0.38 ± 0.12 )
Cor	1.49 ± 0.024 (2.34 ± 0.07)	1.99 ± 0.09 (3.13 ± 0.44)	0.37 ± 0.37 (0.58 ± 0.03)	0.36 ± 0.07 (0.56 ± 0.023)	0.44 ± 0.08 (0.69 ± 0.12)	0.22 ± 0.88 (0.35 ± 0.09)	0.15 ± 0.04 (0.24 ± 0.02)	0.44 ± 0.03 (0.69 ± 0.05)
Phyll	3.20 ± 0.15 (7.60 ± 0.10)	8.51 ± 0.52 (20.2 ± 0.40)	3.70 ± 2.80 (8.80 ± 1.00)	3.2 ± 2.81 (7.6 ± 0.40)	2.38 ± 1.44 (5.68 ± 0.31)	1.40 ± 1.72 (3.34 ± 0.05)	0.08 ± 0.66 (0.20 ± 0.01)	1.54 ± 0.87 (3.68 ± 0.43)
Hypophyll	4.40 ± 0.2 (10.2 ± 0.40)	9.60 ± 0.26 (22.3 ± 0.53)	3.6 ± 1.40 (8.4 ± 1.30)	6.1 ± 2.32 (14.2 ± 1.70)	1.41 ± 0.06 (3.27 ± 0.15)	1.87 ± 0.01 (4.34 ± 0.02)	0.95 ± 0.11 (2.20 ± 0.31)	1.73 ± 0.31 (4.02 ± 0.71)
Ibuprofen	1.40 ± 0.10 (6.60 ± 0.10)	1.98 ± 0.05 (9.57 ± 0.23)						
Aspirin			2.16 ± 0.80 (11.88 ± 4.14)	0.85 ± 0.20 (4.68 ± 2.71)	0.43 ± 0.07 (2.40 ± 0.16)	0.17 ± 0.01 (0.94 ± 0.05)	0.14 ± 0.09 (0.77 ± 0.16)	0.58 ± 0.28 (3.22 ± 0.38)

GA: Gallic acid, EA: Ellagic acid, Ger: Geraniin, Cor: Corilagin, Phyll: Phyllanthin, Hypophyll: Hypophyllanthin, PA: *Phyllanthus amarus*, PU: *Phyllanthus urinaria,* Mal: Malaysia, Ind: Indonesia.

IC_50_ values in *μ*M are in parentheses.

### Phagocytic assay

**Figure 3:**
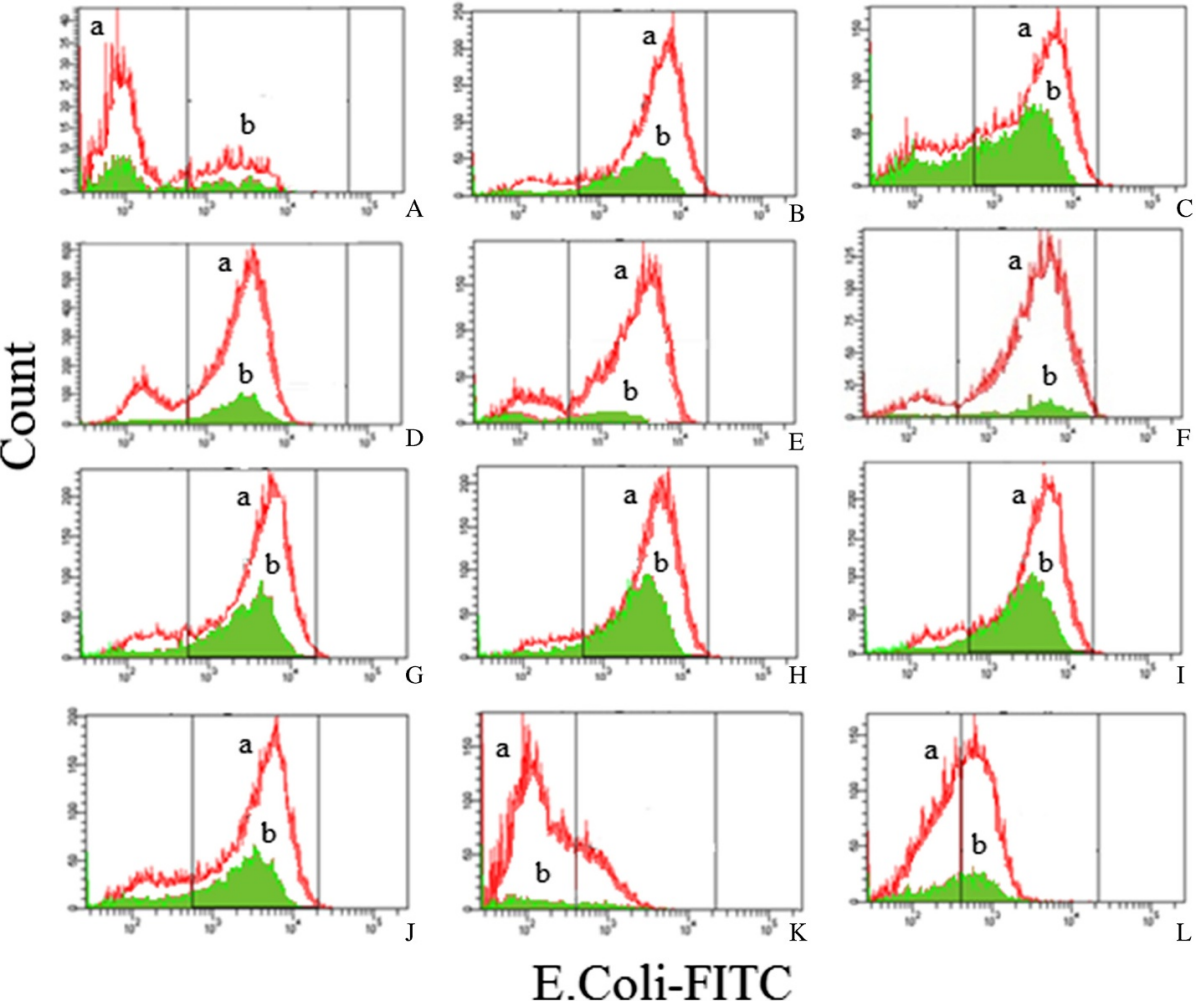
**Representation of** ***E. coli*** **engulfment by (a) Neutrophils and (b) Monocytes treated with plant extracts and their major compounds at 100 μg/mL except phyllanthin and hypophyllanthin at 50 μg/mL. (A)** negative control, **(B)** positive control, **(C)** *P. amarus* (Mal), **(D)** *P. amarus* (Ind), **(E)** *P. urinaria* (Mal), **(F)** *P. urinaria* (Ind), **(G)** Gallic acid, **(H)** Ellagic acid, **(I)** Geraniin, **(J)** Corilagin, **(K)** Phyllanthin, **(L)** Hypophyllanthin.

The ability of PMNs and monocytes to phagocytize opsonized *E. coli* was evaluated by phagotest kit and analyzed by flow cytometry. The *E coli* was opsonized with immunoglobulin and complement of pooled sera. The phagocytic activity was calculated based on the percentage of *E. coli* ingestion by phagocytes. The complement opsonized microbes were recognized by complement surface receptors on leukocytes. The Fab portion of the antibody binds to the antigen, whereas the constant part of the antibody (Fc) binds to an Fc receptor on the phagocyte, facilitating phagocytosis [39]. The extracts of *Phyllanthus amarus* and *P. urinaria* at 100 and 6.25 μg/mL exhibited moderate inhibition of *E. coli* uptake by monocytes but weak effect on neutrophils, as shown in Figure 3 and Table 3. At 100 μg/mL, all plant extracts showed moderate engulfment inhibitory activity with percentage of phagocytizing cells ranging from 53.70 to 66.68% for monocytes. The engulfment inhibitory activity at normal condition at 37°C was used as a positive control and normal condition at 0°C as negative control (Table 3). Except for phyllanthin and hypophyllanthin, all pure compounds did not show significant activity on the bacterial engulfment by human leukocytes. Phyllanthin at 50 μg/mL exhibited the highest engulfment inhibitory activity with percentage of phagocytizing cells of 14.2 and 27.1% for PMNs and monocytes, respectively. Phyllanthin might be the major contributor of phagocytic activity of the plant extracts. The results suggest that the moderate inhibitory of intake complement and opsonized *E. coli* of the compounds might be due to the inhibition of the complement surface receptors on leukocytes.

**Table 3:** **Percentage of phagocytic activity (%) of neutrophils and monocytes at various concentrations of** ***Phyllanthus amarus and P. urinaria*** **extracts and their major compounds (mean ± SEM,** ***n*** **=3)**

Sample (μg/mL)	Neutrophils				Monocytes			
	100	50	6.25	3.125	100	50	6.25	3.125
PA (Mal)	75.60 ± 1.82		88.20 ± 1.17		53.70 ± 0.15		65.68 ± 0.15	
PA (Ind)	81.65 ± 2.21		89.29 ± 2.67		66.68 ± 3.16		74.60 ± 2.76	
PU (Mal)	78.13 ± 2.07		89.60 ± 0.98		59.08 ± 1.66		68.80 ± 1.66	
PU (Ind)	73.30 ± 3.32		79.40 ± 1.07		61.86 ± 2.81		72.22 ± 3.02	
GA	88.60 ± 1.52		95.90 ± 1.97		76.50 ± 2.18		77.80 ± 1.71	
EA	85.20 ± 1.19		88.18 ± 2.13		75.50 ± 1.97		67.10 ± 2.31	
Ger	76.00 ± 3.05		80.80 ± 1.06		76.08 ± 3.16		84.90 ± 0.91	
Cor	85.50 ± 2.82		86.10 ± 0.32		80.70 ± 1.51		85.38 ± 1.04	
Phyll		14.20 ± 1.08		34.91 ± 0.09		27.10 ± 0.55		52.10 ± 0.68
Hypophyll		49.11 ± 1.04		80.50 ± 0.87		64.6 ± 0.51		67.0 ± 1.07
Positive control	91.60 ± 1.15				84.6 ± 4.84			

See Table 2 for legends.

### Inhibition of CD18 expression assay

The effects of the *P. amarus* and *P. urinaria* extracts on β2 integrins (CD18) expression on PMNs and monocytes are shown in Table 4. The inhibition of CD18 expression by plant extracts and compounds was tested with the aid of flow cytometry. Except foe phyllanthin and hypophyllanthin, the plant extracts and pure compounds possessed weak inhibition of CD18 expression on human leukocytes. Phyllanthin and hypohyllanthin depicted significant inhibition at 50 μg/mL with percentage of CD18 expression of 67.17% and 61.56% for PMNs and monocytes, respectively. The expression of CD18- containing integrins is essential for the normal functioning of neutrophil *in vivo*. It plays an important role of cytotoxic killing by T cells. On the other hand, the binding of human leukocyte integrins (CD11a/CD18, CD11b/CD18 and CD11c/CD18) to lipopolysaccharide (LPS), a bacterial endotoxin, triggers deleterious systemic inflammatory responses when released into blood circulation, causing organ damage [40, 41]. The suppression of LPS binding site on human leukocytes may reduce the progress of inflammatory processes by inhibiting the expression of these integrins.

**Table 4:** **Percentage of CD18 expression (%) on neutrophils and monocytes at various concentrations of** ***Phyllanthus*** **extracts and their major compounds**

Sample (μg/mL)	Neutrophils				Monocytes			
	100	50	6.25	3.125	100	50	6.25	3.125
PA (Mal)	79.62 ± 1.82		87.10 ± 1.23		70.79 ± 2.13		85.68 ± 1.10	
PA (Ind)	81.65 ± 2.21		89.29 ± 2.67		62.39 ± 1.87		89.29 ± 1.51	
PU (Mal)	82.90 ± 1.08		90.62 ± 1.80		76.20 ± 1.54		88.98 ± 2.76	
PU (Ind)	87.15 ± 3.39		93.20 ± 3.39		85.18 ± 0.51		85.41 ± 1.69	
GA	81.33 ± 1.80		88.77 ± 2.41		68.60 ± 1.20		76.50 ± 2.18	
EA	86.14 ± 2.01		90.18 ± 1.07		62.20 ± 0.57		77.10 ± 1.99	
Ger	89.40 ± 1.21		93.10 ± 1.23		82.90 ± 1.71		88.40 ± 1.75	
Cor	92.10 ± 1.02		95.80 ± 0.98		79.51 ± 1.10		85.38 ± 1.32	
Phyll		67.17 ± 1.34		79.20 ± 2.92		61.56 ± 0.98		72.21 ± 1.26
Hypophyll		74.70 ± 0.09		80.90 ± 1.65		74.42 ± 0.77		75.15 ± 1.48
Positive control	93.93 ± 0.54				84.79 ± 0.55			

See Table 2 for legends.

(Mean ± SEM, *n* =3).

### Chemiluminescence assay

**Figure 4:**
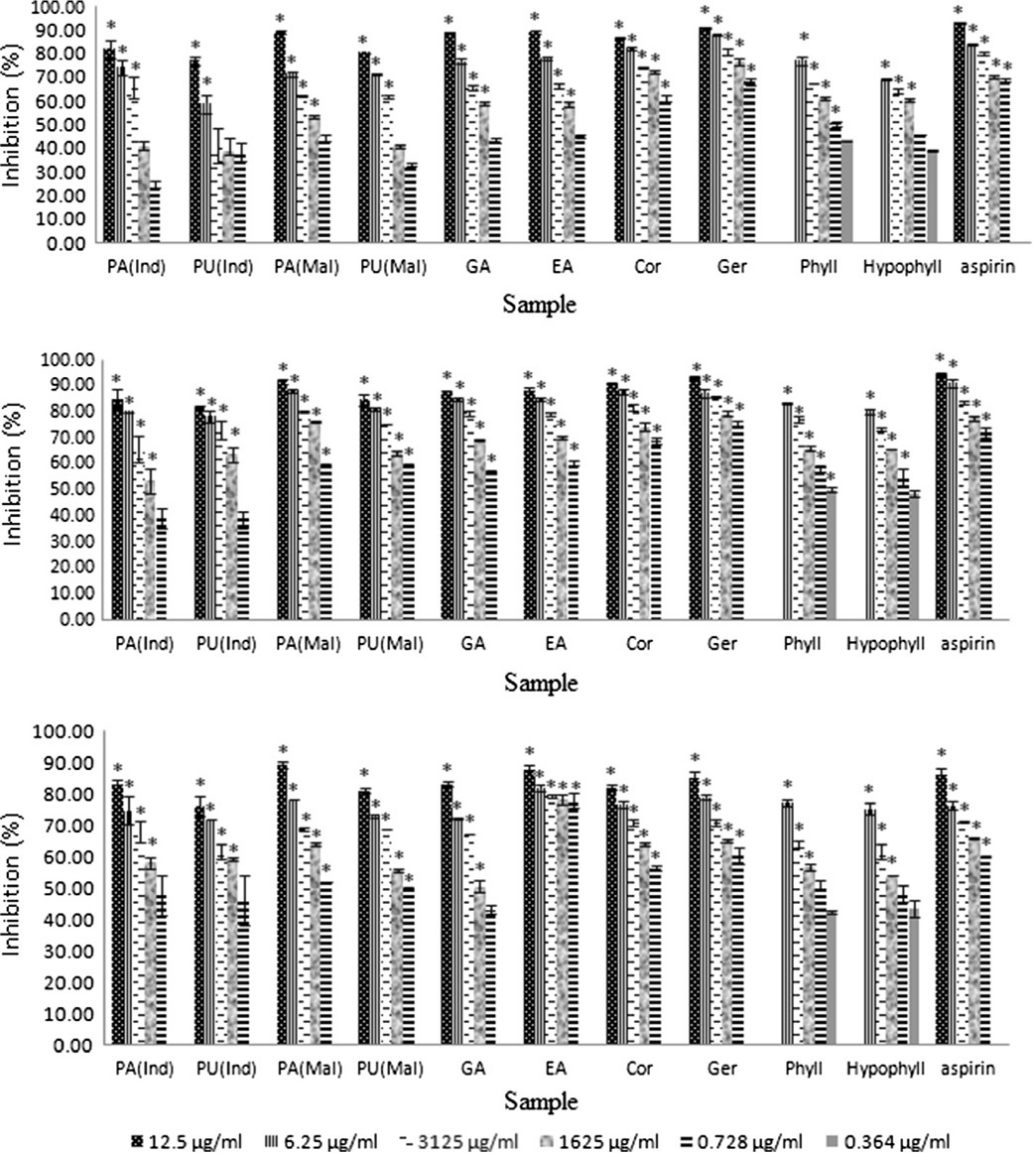
**Percentage inhibition (%) of ROS inhibitory activity of** ***Phyllanthus*** **sp. and their major compounds on PMA stimulated whole blood (a), PMNs (b) and MNCs (c) assayed by luminol amplified chemiluminescence.** Data are mean ± SEM (*n* =3). Significance of differences with respective control: *P< 0.05. See Table 2 for legends.

Preliminary screening of the plant extracts on whole blood showed that *P. amarus* and *P. urinaria* from Malaysia exhibited high inhibitory activity for luminol-enhanced chemiluminescence with IC_50_ values of 0.95 and 1.52 μg/mL, respectively, for zymosan induced and 1.24 and 1.98 μg/mL, respectively, for PMA induced (Table 2 and Figure 4). The Malaysian *Phyllanthus* samples were also more potent than the Indonesian samples against PMNs and monocytes. The extracts of *P. amarus* possessed higher inhibitory activity compared to *P. urinaria*. This might be associated with the presence of high amount of bioactive phenolic compounds in *P. amarus* compared to *P. urinaria*. Geraniin and corilagin exhibited IC_50_ values of 0.36 and 0.58 μM, respectively, in zymosan stimulated whole blood, and 0.21 and 0.35 μM, respectively, in PMA stimulated whole blood. Their IC_50_ values were lower than those of aspirin, indicating that they were more potent than the positive control. Aspirin was used as the positive control based on a previous report that the drug inhibited luminol-enhanced chemiluminescence of human leukocytes [42].

The extracts of *Phyllanthus* species and their major components were further investigated for their effects on the oxidative burst of PMA or zymosan stimulated PMNs and monocytes. The plant extracts from both countries and pure compounds showed significantly high inhibition of ROS production from zymosan and PMA stimulated leukocytes. Geraniin and corilagin exhibited exceptionally strong inhibition on ROS activity against PMNs and monocytes. The IC_50_ values of geraniin and corilagin were 0.18 and 0.56 μM, respectively, for zymosan induced and 0.13 and 0.24 μM, respectively for PMA induced PMNs. Their IC_50_ values were similarly much lower than those of aspirin against monocytes, indicating that they were more potent than the positive control. Figure 4 shows the percentage inhibition of oxidative burst of the *Phyllanthus* species and the pure compounds on PMA induced leukocytes, depicting a dose-dependent relationship.

It is increasingly proposed that ROS plays a key role in structural alteration of DNA, dysregulation of cytoplasmic and nuclear signal transduction and effect the activity of the proteins and genes that respond to stress and which act to regulate the genes that are related to cell proliferation, differentiation and apoptosis pathways. ROS is produced by phagocytes in response to a variety of stimulators for bacterial killing and amplification of inflammatory response. The NADPH oxidase complex plays essential role in ROS production. The activation of NADPH oxidase is induced by receptor ligation and engulfment of microbial pathogens, chemical or various stimuli, such as PMA, fMLP, or zymosan. In this study ROS generation was assessed upon stimuli with PMA which crosses the cellular membrane and binds to protein kinase C independent of cell receptor interaction and serum opsonized zymosan which stimulates neutrophils via their surface complement receptor (CR3) [43]. The present study indicates that similar inhibitory activity of ROS generation was noticed in phagocytes irrespective of the type of stimuli used. This clearly shows that *P. amarus* and *P. urinaria* have the capacity to inhibit ROS production through two different pathways.

Besides the defensive roles of phagocytes against infectious microbes, the phagocyte-microbe interactions between excessively or inappropriately deployed can damage host tissues and contribute to the pathogeny of various immune and nonimmune chronic inflammatory diseases. Therefore, the strong inhibition of the major compounds of *P. amarus* and *P. urinaria* on phagocytic activity of human phagocytes indicates that the extracts and their major constituents have the potential to be developed into agents for the treatment of a variety of disorders including inflammation.

## Conclusions

The HPLC analysis methods proposed in this study enable identification and quantification of all major compounds found in the 80% ethanol extracts of *P. amarus* and *P. urinaria*. The HPLC quantification analysis indicated the presence of high amount of phenolic compounds in the plant extracts obtained from both Malaysia and Indonesia. The *in vitro* studies revealed that, the plant extracts, especially Malaysian *P. amarus* exhibited potent inhibitory activity of ROS generation and chemotactic activity of phagocytes stimulated by different stimuli. The results suggest that the immune regulatory action of the plant extracts act through various pathways. The phenolic compounds, geraniin, corilagin and ellagic acid demonstrated strong inhibition against the leukocytes. Geraniin and corilagin also exhibited exceptionally strong inhibition on ROS activity against PMNs for zymosan induced PMA induced leukocytes. In contrast, the phenolic compounds did not show significant effect on phagocytic activity and β2 integrins expression of phagocytes. The inhibitory activity possessed by plant extracts at these steps could be due the contribution of phyllanthin and hypophyllanthin which exhibited potent inhibitory action on both phagocytic and CD18 expression of phagocytes. Thus, the *in vitro* study suggest that the presence of high levels of the major compounds of *P.amarus* and *P.urinaria* reported in this study could be the major contributors to the strong immunomodulatory effect of the plant.
